# Bridging research and practice in conservation

**DOI:** 10.1111/cobi.13732

**Published:** 2021-06-04

**Authors:** Andrew N. Kadykalo, Rachel T. Buxton, Peter Morrison, Christine M. Anderson, Holly Bickerton, Charles M. Francis, Adam C. Smith, Lenore Fahrig

**Affiliations:** ^1^ Department of Biology Carleton University Ottawa Ontario Canada; ^2^ EcoEcoAnalysis Ottawa Ontario Canada; ^3^ Department of Natural Resource Sciences McGill University Sainte‐Anne‐de‐Bellevue Quebec Canada; ^4^ Parks Canada Ottawa Ontario Canada; ^5^ Canadian Wildlife Service Environment and Climate Change Canada Ottawa Ontario Canada

**Keywords:** environmental evidence, evidence‐based conservation, evidence‐informed decision making, knowledge broker, knowledge exchange, knowledge translation, natural resource management, research‐implementation gap, brecha en la implementación de la investigación, conservación basada en la evidencia, evidencia ambiental, intercambio de conocimiento, intermediario de conocimiento, manejo de recursos naturales, toma de decisiones guiada por la evidencia, traducción del conocimiento, 基于证据的保护, 知识交流, 知识中介, 知识转化, 环境证据, 自然资源管理, 研究与实施的差距, 循证决策

## Abstract

Calls for biodiversity conservation practice to be more evidence based are growing, and we agree evidence use in conservation practice needs improvement. However, evidence‐based conservation will not be realized without improved access to evidence. In medicine, unlike in conservation, a well‐established and well‐funded layer of intermediary individuals and organizations engage with medical practitioners, synthesize primary research relevant to decision making, and make evidence easily accessible. These intermediaries prepare targeted evidence summaries and distribute them to practitioners faced with time‐sensitive and value‐laden decisions. To be effective, these intermediaries, who we refer to as *evidence bridges*, should identify research topics based on the priorities of practitioners; synthesize evidence; prepare and distribute easy‐to‐find and easy‐to‐use evidence summaries; and develop and maintain networks of connections with researchers and practitioners. Based on a review of the literature regarding evidence intermediaries in conservation and environmental management, as well as an anonymous questionnaire searching for such organizations, we found few intermediaries that met all these criteria. Few evidence bridges that do exist are unable to reach most conservation practitioners, which include resource managers in government and industry, conservation organizations, and farmers and other private landowners. We argue that the lack of evidence bridges from research to practitioners contributes to *evidence complacency* and limits the use of evidence in conservation action. Nevertheless, several existing organizations help reduce the gap between evidence and practice and could serve as a foundation for building additional components of evidence bridges in conservation. Although evidence bridges need expertise in research and evidence synthesis, they also require expertise in identifying and communicating with the community of practitioners most in need of clear and concise syntheses of evidence.

Article Impact Statement: Evidence‐based conservation will not be realized without improved access to evidence. We call for intermediary evidence bridges.

## Abstract 2

*Article impact statement*: Evidence‐based conservation will not be realized without improved access to evidence. We call for intermediary evidence bridges.

## Abstract (es)

Vinculación entre la Investigación y la Práctica en la Conservación

**Resumen:**

Cada vez existen más peticiones para que las prácticas de conservación de la biodiversidad estén más basadas en evidencias, además de que apoyamos la idea de que el uso de evidencias en la práctica de la conservación necesita mejorar. Sin embargo, la conservación basada en la evidencia no se logrará sin un acceso mejorado a las evidencias. En la medicina, no como en la conservación, un estrato bien establecido y financiado de individuos y organizaciones intermediarias interactúan con los médicos, sintetizan las investigaciones primarias relevantes para la toma de decisiones y hacen que las evidencias sean de fácil acceso. Estos intermediarios preparan resúmenes de evidencias específicas y los distribuyen a los médicos que enfrentan decisiones urgentes y muy valiosas. Para que sean efectivos, estos intermediarios, a quienes nos referimos como *puentes de evidencias*, deben poder identificar los temas de estudio con base en las prioridades de los practicantes, sintetizar evidencias, preparar y distribuir resúmenes fáciles de encontrar y fáciles de usar, y desarrollar y mantener redes de conexiones con los investigadores y los practicantes. Con base en una revisión de la literatura correspondiente a los intermediarios de evidencias en la conservación y el manejo ambiental, así como en un cuestionario anónimo que busca a dichas organizaciones, encontramos a pocos intermediarios que cumplieran con estos criterios. Los pocos puentes de evidencias que existen no son capaces de llegar a la mayoría de los practicantes de la conservación, los cuales incluyen a los gestores de recursos en el gobierno y en la industria, a las organizaciones de conservación y a los agricultores y otros terratenientes privados. Argumentamos que la falta de puentes de evidencia entre los investigadores y los practicantes contribuye a la *indulgencia de evidencias* y limita el uso de evidencias en las acciones de conservación. Sin embargo, varias organizaciones existentes ayudan a reducir la brecha entre la evidencia y la práctica y podrían funcionar como base para la construcción de componentes adicionales para los puentes de evidencia en la conservación. Aunque los puentes de evidencias necesitan experiencia con la investigación y con la síntesis de evidencias, también requieren experiencia con la identificación de y comunicación con la comunidad de practicantes que más necesitan una síntesis clara y concisa de la evidencia.

## 摘要

对于生物多样性保护实践应更加注重证据的呼声已越来越高, 我们也同意在保护实践中需要改进证据的使用。然而, 如果不能获取更好的证据, 也将无从实现基于证据的保护。与保护领域不同的是, 在医学领域中, 由已确立并资金充足的中介人或中介组织与医务人员合作, 来整合与决策相关的第一手研究资料, 从而使这些研究证据更易于获取。这些中介机构会准备有针对性的证据摘要, 并将其分发给需要进行较强时效性、主观性决策的医务人员。为了提高效率, 中介机构 (我们称之为“证据桥梁”) 应根据医务人员的优先排序来确定研究课题、整合证据、准备和分发易获得且易使用的证据摘要, 并发展和保持研究者与医务工作者的联系网络。基于对保护及环境管理领域证据中介研究的文献调研, 以及此类机构的匿名问卷搜索, 我们发现很少有中介能达到全部标准。现有的少量证据桥梁无法影响到大多数包括政府和行业的资源管理人员、保护组织、农民及其他私人土地所有者在内的保护实践者。我们认为, 缺乏从研究到实践者的证据桥梁会导致“证据自满”, 并限制保护行动中的证据使用。尽管如此, 已有的一些组织能帮助缩小证据与实践之间的差距, 并可作为建立保护证据桥梁额外组成部分的基础。建立证据桥梁不仅要有研究和证据整合的专门知识, 还要有识别出最需要清晰简明的整合证据的实践者群体并与之沟通的专门知识。 **【翻译: 胡怡思; 审校: 聂永刚】**

## INTRODUCTION

Salafsky et al. (2019) added their voices to a growing call for biodiversity conservation and environmental management (hereafter conservation) to be more evidence‐based (Nguyen et al., 2017; Pullin, 2012; Pullin & Knight, 2001; Pullin & Knight, 2003; Sutherland et al., 2004). Although we agree with Salafsky et al. (2019) that evaluation and use of evidence in conservation practice can be improved, evidence‐based conservation cannot be realized without also improving awareness of and access to centralized, user‐friendly syntheses of evidence. By *evidence*, we mean scientifically generated relevant information or knowledge used to assess hypotheses related to a question of interest (Salafsky et al., 2019). We suggest that easy access to user‐friendly evidence is rare in conservation, contributing to knowledge‐action gaps in which the available science is not widely used (Appendix S1). A lack of accessible evidence can lead to “evidence complacency” in which, despite the availability of evidence, it is not sought or used to make conservation decisions (Sutherland & Wordley, 2017).

Salafsky et al. (2019) contrast decision making in conservation with decision making in medicine, arguing that in medicine, decisions are more strongly grounded in evidence. They suggest that this difference is largely because medicine is a more mature discipline, with well‐defined situations, whereas conservation involves complex and messy situations. We agree that these are important factors, but here, we highlight an additional critical difference:––medicine has a well‐established and well‐funded layer of intermediary individuals and organizations that synthesize primary research and make it easily accessible to medical practitioners in ways that support time‐sensitive decisions. These intermediaries prepare evidence summaries and syntheses, and distribute them in an appropriate manner to inform practitioners’ decisions, thus facilitating direct access to evidence (see Figure 4 in Salafsky et al. [2019]). Hence, they form a bridge linking research conducted by scientists to the practitioners who need information to improve their actions (Haynes et al., 1995; Lomas, 1997b; Lomas, 2007). In the health professions, these intermediaries are known as knowledge translators.

We included all knowledge translation professionals and activities under the term *evidence bridges* and suggest that these are generally absent or insufficient in conservation. We considered the need for widely recognized and easily accessible evidence bridges so that conservation practitioners can make timely and informed decisions about possible actions on the ground. In our view, conservation practitioners are analogous to medical practitioners and include resource managers in government or industry, conservation organizations, consultants, and farmers and other private landowners.

We examined the current use of evidence and major barriers to the use of that evidence in conservation; reviewed the key elements of knowledge translation and exchange in medicine and conservation; defined the key roles and properties required to form effective evidence bridges in conservation; contrasted evidence bridges with organizations that synthesize conservation evidence but are not fully formed, stand‐alone evidence bridges; reviewed existing organizations that partially fulfill the role of evidence bridges in conservation and what they could do to become full evidence bridges; reviewed the roles of researchers and practitioners and why neither group is well equipped to form evidence bridges; examined the role of values in conservation decisions; and devised a model for how evidence bridges could be funded and how a possible college for conservation decision making could be the basis of a professional community to support evidence‐based conservation.

## CURRENT USE OF EVIDENCE IN CONSERVATION AND MAJOR BARRIERS

To examine the current status of evidence use in conservation, we reviewed the scientific literature for studies that used surveys or interviews to understand how conservation practitioners use evidence (Table 1; methods in Appendix S2). We found 19 relevant studies that, taken together, suggest that evidence, especially peer‐reviewed science, is rarely the first or most widely used or the most valued source of knowledge or information considered in conservation decisions. The available studies were primarily limited to interviews of resource managers. Other kinds of practitioners, such as farmers and other private landowners, may access and use evidence differently in conservation decision making.

**TABLE 1:** A summary of articles investigating the use of evidence in conservation decision making (ordered chronologically)

References	Potential evidence users	Use of evidence in decision making	Major barriers to using scientific evidence*
Morrison‐Saunders and Bailey (2003)	environmental impact assessment practitioners (Australia)	Although science was perceived to provide the basis for baseline data collection, impact prediction, and mitigation design, it was seen as less important during decision making and ongoing project management.	organizational capacity, resources, and finance; social, political, and economic context of the decision
Pullin and Knight (2005); Pullin et al. (2004)	conservation management plan compilers (United Kingdom and Australia)	Most frequent evidence sources were existing management plans (60%), expert opinion (49%), secondary literature (47%), and accounts of traditional management practices (46%). Less frequent sources were published scientific papers (23%). Those that always used published scientific papers were in the minority (8% U.K. and 17% Australia), and 12% of U.K. compilers said they never accessed the primary literature.	accessibility of the evidence; organizational capacity, resources, and finance
Sutherland et al. (2004)	wetland site managers (United Kingdom)	In total, 77% of sources were anecdotal (“common sense,” personal experience, and speaking to other managers), whereas only 2% were based upon verifiable scientific evidence.	not assessed
Cook et al. (2010)	protected area managers (Australia)	Around 60% of conservation management decisions rely on experience‐based information.	accessibility of the evidence
Young and Van Aarde (2011)	protected area managers (South Africa)	Most managers base decisions on experience‐based information. Only 28% of managers developed objectives, 30% identified issues, 8% selected management methods, 30% selected the conservation objective, and 5% selected the intervention method, according to science‐based information.	accessibility of the evidence; relevance and applicability of the evidence; quality, credibility, and legitimacy of the evidence; researcher communication and dissemination skills
Bayliss et al. (2012)	practitioners and stakeholders working with invasive species (United Kingdom)	The most widely used information sources were general internet searches, invasive species websites, and colleague knowledge (used by 87.8% of respondents).	accessibility of the evidence
Cook et al. (2012)	protected area managers (Australia)	While valuing empirical evidence most highly for their decisions, managers reported having poorer access to these data than other information or knowledge, such as experience‐based anecdotes, management plans, and legislation, which they viewed as less valuable.	accessibility of the evidence
Cvitanovic et al. (2014)	marine protected area management plans (Australia, Kenya, and Belize)	Most management plan information sources were commissioned technical reports (52%), followed by local government reports (23%). Primary science was the third most frequently used knowledge source (14%). Information was not available on whether recommendations in technical reports and government documents were based on peer‐reviewed science or personal judgment.	accessibility of the evidence; relevance and applicability of the evidence; researcher communication and dissemination skills
Matzek et al. (2014)	land managers and restoration professionals (United States)	Practitioners rely on their own experience, and generally do not read the peer‐reviewed literature, which they regard as only moderately useful. Less than half of managers who do research carry out experiments conforming to the norms of hypothesis testing, and their results are not broadly disseminated.	accessibility of the evidence; practitioner skills for understanding and using science practitioner time to find and read evidence; relevance and applicability of the evidence
Addison et al. (2015)	marine protected area management agencies (Australia)	Even when long‐term monitoring results are available, management agencies are not using them for quantitative condition assessment. Instead, many agencies conduct qualitative condition assessments, where monitoring results are interpreted using expert judgment only.	not assessed
Ntshotsho et al. (2015)	natural resource managers (South Africa)	Intuition was a common determinant of what, where, and how to clear invasive alien plants, thus emerging as a particularly strong factor in the location of clearing projects. Only 3 of the 7 documents analyzed made specific reference to scientific literature.	social, political, and economic context of the decision
Cvitanovic et al. (2016)	Ningaloo Marine Park managers and decision‐makers (Australia)	Although the Ningaloo Research Program generated expansive and multidisciplinary science outputs directly relevant to the management of the Ningaloo Marine Park, decision‐makers are largely unaware of this knowledge and little has been integrated into decision‐making processes.	accessibility of the evidence; practitioner awareness of the literature; researcher–practitioner links; researcher communication and dissemination skills
Young et al. (2016)	government fisheries managers and scientists, stakeholders (Canada)	The percentage of respondents consulting scientific publications as a first source of information is 9% and 13% for government employees and stakeholders, respectively.	accessibility of the evidence
Giehl et al. (2017)	protected area managers (Brazil)	Managers most frequently made decisions based on their personal experience, with scientific evidence being used relatively infrequently.	accessibility of the evidence; practitioner skills for understanding and using science
Artelle et al. (2018)	wildlife management agencies (United States and Canada)	For most species in most jurisdictions, natural resource management lacked the basic elements of a scientific approach, that is, measurable objectives, evidence, transparency, and independent review.	social, political, and economic context of the decision
Koontz and Thomas (2018)	ecosystem management state agency (United States)	Ecosystem management plans contained no references to peer‐reviewed scientific journal articles in the text. The most common documents in summary tables were gray literature.	not assessed
Lemieux et al. (2018)	protected area managers (Canada)	Information produced by staff within the organizations is given priority over other forms of empirical evidence, such as Indigenous knowledge and peer‐reviewed literature.	organizational capacity, resources, and finance; practitioner time to find and read evidence; researcher–practitioner links
Fabian et al. (2019)	professionals in government, NGOs, national parks, private consultancies, forestry (Switzerland)	Experience‐based information sources, such as personal experience and direct exchange with colleagues and experts, are more important than evidence‐based sources, such as guidelines, specialized journals, and textbooks targeted to professionals. Articles from international scientific journals are hardly ever consulted.	accessibility of the evidence; practitioner time to find and read evidence; relevance and applicability of the evidence

* Major barriers to using scientific evidence were categorized according to the typology developed by Walsh et al. (2019).

Decision‐makers relied heavily on judgment and experience, including personal experience, anecdotes, and personal contacts with colleagues and experts––without clear links to evidence (Table 1). When evidence existed, it was often not in a form suitable for use by practitioners (Table 1). Using the typology developed by Walsh et al. (2019), we found the most common barriers to use of scientific evidence were accessibility of the evidence (12 studies); relevance and applicability of the evidence (4 studies); organizational capacity, resources, and finances (4 studies); time required to find and read evidence (3 studies); and researcher communication and dissemination skills (3 studies).

Several studies, through interviews with conservation practitioners, documented the need to identify and engage effective intermediaries to improve the exchange of evidence (Cvitanovic et al., 2016; Nguyen et al., 2018; Reed et al., 2014).

## KNOWLEDGE TRANSLATION, KNOWLEDGE EXCHANGE, AND EVIDENCE INTERMEDIARIES

In the health professions, the term *knowledge translation* (Graham et al., 2006; Grimshaw et al., 2012) is generally used to describe the role of knowledge translators (or brokers)––evidence intermediaries who link research producers and end users. Formally, *knowledge translation* is defined, for example, by the Canadian Institutes of Health Research (https://cihr‐irsc.gc.ca/e/193.html) as a dynamic and iterative process that includes synthesis, dissemination, exchange, and ethically sound application of knowledge to improve health, provide more effective health services and products, and strengthen the health care system (Graham et al., 2006; Straus et al., 2009).

The origins of knowledge translation can be traced to informal networks linking academic researchers with the German dye (late 1800s) and agricultural industries (1906) (Lomas, 2007). The concept of knowledge translation in health emerged in the 1990s, when knowledge producers “pushed” their research messages onto end‐users (Peprah, 2020). Clinical epidemiologists, clinicians who see patients and do research, are considered the first medical knowledge brokers from this period (i.e., early 1990s) (Lomas 2007). Although the practice of knowledge translation in health has recently expanded to more organizations and health professions, knowledge translators typically do the translation (synthesis), making first contact with practitioners and disseminating information to them (Lomas 2007). It is not often the other way around (i.e., practitioners initially engage with or seek out knowledge translators).

In conservation and environmental science, the similar concept of knowledge exchange has gained prominence. Fazey et al. (2012) define it as “processes that generate, share, and/or use knowledge through various methods appropriate to the context, purpose, and participants involved.” *Boundary organizations* or *spanners*, *bridging organizations*, and *knowledge brokers* are all related terms that are commonly applied but have varied meanings. As in medicine, the evidence intermediaries in conservation aim to enable effective knowledge exchange. However, conservation intermediaries (as opposed to those in medicine) are more varied and ill‐defined (Bednarek et al., 2018; Crona & Parker, 2012; Gustafsson & Lidskog 2018) and, we argue, insufficient in number and underdeveloped in role and function for translating research into practice.

## KEY ROLES AND PROPERTIES OF EVIDENCE BRIDGES

**FIGURE 1:**
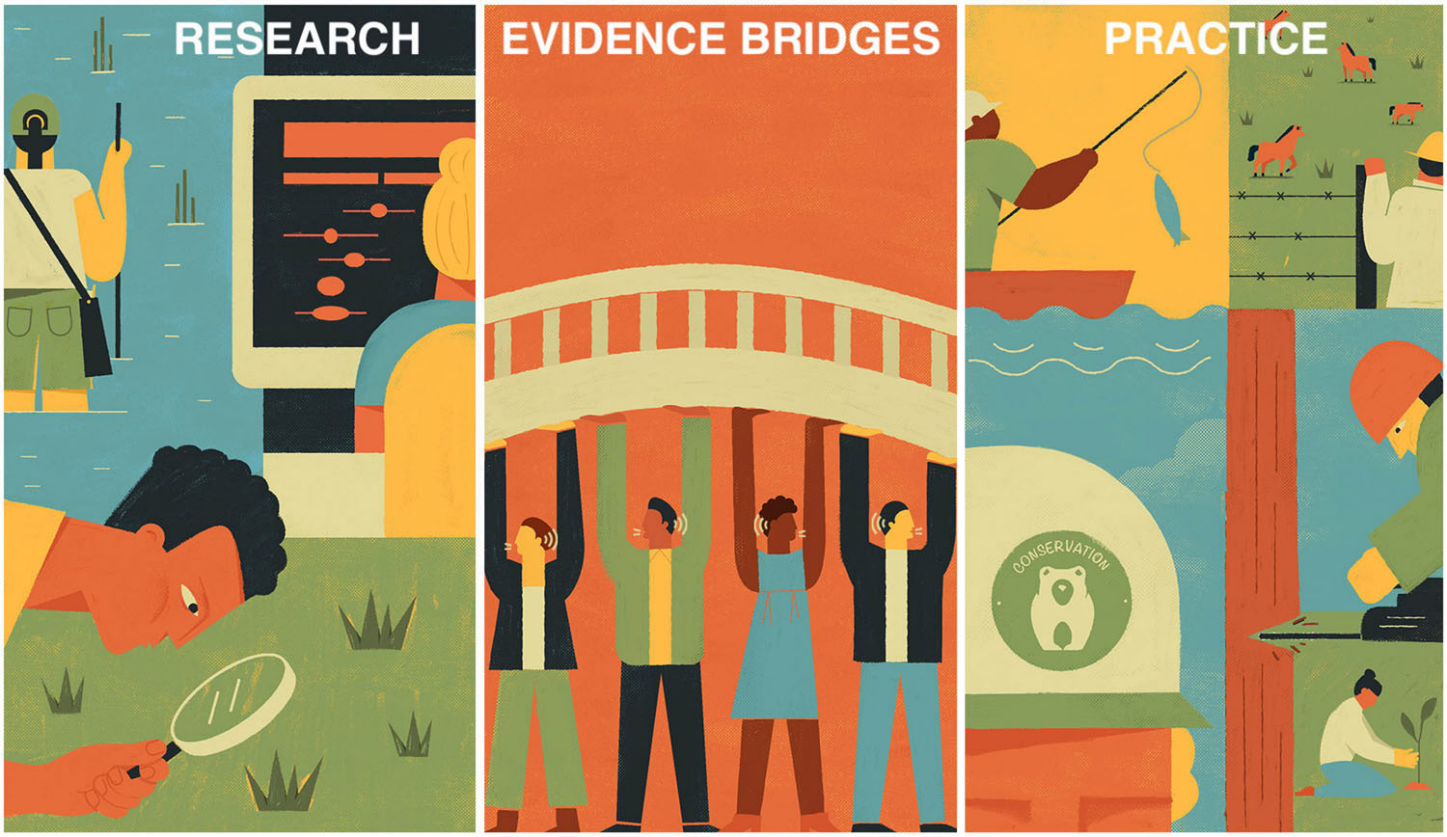
Conceptualization of evidence bridges who identify research topics based on input from practitioners and facilitate the translation of research into conservation practice (e.g., forest management, sustainable fishing, water management, etc.)

Evidence bridges are knowledge brokers (Cvitanovic et al., 2015; Farwig et al., 2017; Meyer, 2010) who facilitate the exchange of knowledge between and among researchers and practitioners (Figure 1). A set of key roles and properties for effective evidence bridges based on our review of evidence bridges in medicine and the knowledge broker literature is in Table 2.

**TABLE 2:** Desirable features and functions for evidence bridges between research and practice in conservation^a^

**Mandate**	
Professional individuals or organizations with a mandate to act as full‐time evidence intermediaries (5,13) between science and practice (i.e., they support conservation decisions of practitioners in their on‐the‐ground/in‐the‐water actions).	
**Skills and training**	
Strong communication skills and training in both science and decision making to identify relevant topics for synthesis through consultation with practitioners, and to appropriately translate research findings for practitioners (1,3,5,10,11)	
**Synthesizing evidence**	
Comprehensively search for, identify, and collect available research evidence (4,5,6,18)	Produces^b^ evidence syntheses that are directly useable by practitioners during on‐the‐ground decision making
Assess the evidence for quality and quantity (4,5,11,18)	
Consider the effectiveness of a given intervention and other factors that might affect investment in the intervention (e.g., cost, availability of alternatives, and effect of not acting) (16)	
Identify, interpret, translate, and summarize key messages for different practitioner audiences relevant to their needs and questions (4,5,7,11,12,18)	
Produce “brokered knowledge” (13): Tailor messages to the local context to ensure their relevance and suitability (5,11,14,18)	
**Preparing evidence summaries and tools**	
Produce or assemble brief, plain language research summaries, that is, concise, targeted, and relevant tools, algorithms, synopses, and guidelines (4,12)	Produces evidence syntheses that are directly useable by practitioners during on‐the‐ground decision making^b^
Produce or assemble evidence summaries and tools that are practitioner‐focused: directly useable by practitioners when making decisions on the ground (4,5,7)	
Update evidence summaries and tools regularly from the current state of knowledge (4)	
Produce or assemble evidence summaries and tools that are primarily noncommissioned and therefore independent of economic or other interests that may affect or reasonably be perceived to affect objectivity and independence (8)	Produces evidence syntheses that are primarily non‐commissioned, avoiding or restricting economic or other interests that may affect objectivity and independence
Produce or assemble evidence summaries and tools that address a wide range of research topics that are based on the priorities of practitioners rather than those of the scientific community (7,9)	Identifies research topics sourced from the priorities of practitioners
**Disseminating evidence**	
Distribute evidence syntheses through face‐to‐face exchanges (12)	Distributes evidence syntheses through face‐to‐face exchanges
Distribute easy‐to‐find and easy‐to‐use evidence syntheses to practitioners (7,12,14)	Distributes easy‐to‐find and easy‐to‐use evidence syntheses to practitioners
**Networking**	
Develop and maintain networks of connections among and between researchers and practitioners, developing a mutual understanding of goals and cultures (3,5,6,7,,13,14,17)	Develops and maintains networks of connections among and between researchers and practitioners
Consult practitioners regularly regarding their evidence needs and in doing so inform future research (5,7)	
Identify and communicate practitioner needs (based on the priorities of practitioners) for which research solutions are required to the research community (4,5,7)	
Build trust with a broad base of both research and practitioner communities to make evidence visible and widely sought (5,13,17)	
Promote a culture that values the use of the best available evidence in practice (2,3,5,13)	
**Operations**	
Operate as a neutral third party, independent of financial interests from researchers and decision‐makers (2,15)	Produces evidence syntheses that are primarily non‐commissioned, avoiding or restricting economic or other interests that may affect objectivity and independence

^a^ References: 1, Cook et al. (2013a); 2, Cvitanovic et al. (2015); 3, Cvitanovic et al. (2016); 4, Dicks et al. (2014); 5, Dobbins et al. (2009); 6, Farwig et al. (2017); 7, Gagnon (2011); 8, Karlsson and Takahashi (2017); 9, Knight et al. (2008); 10, Lomas (1997a); 11, Lomas (1997b); 12, Lomas (2007); 13, Meyer (2010); 14, Michaels (2009); 15, Nguyen et al. (2017); 16, Segan et al. (2011);  17, Stern et al. (2021).; 18, Yost et al. (2014)

^b^ Text in this column relates to the most salient and necessary criteria linked to Appendix S6.

Effective evidence bridges interact with primary researchers and practitioners and seek wide recognition and respect in the relevant practitioner communities. They provide existing evidence in easy‐to‐find and easy‐to‐use formats for practitioners. These include concise, targeted, and relevant tools, algorithms, synopses, and guidelines that address a wide range of practitioner‐directed conservation goals, from local concerns (e.g., managing a wetland) to national or global issues (e.g., halting bird population declines, reducing pesticide use, or developing sustainable fisheries). They also consult practitioners regularly regarding their evidence needs and in doing so inform future research. They operate as a third party, independent of financial or other interests of researchers and decision‐makers that could affect objective evidence synthesis and communication.

Evidence bridges are professional individuals or organizations with a mandate to act as intermediaries between science and practice (i.e., they support decisions of conservation practitioners regarding their actions). We distinguish evidence bridges from *boundary spanners*, who focus primarily on the science–policy interface, often as mediators in disputes, posing potential solutions, and deciding on courses of action (Bednarek et al., 2018; Cash, 2001; Cash et al., 2003; Guston, 1999; Guston, 2001; Posner & Cvitanovic, 2019; Safford et al., 2017). We also distinguish evidence bridges from *bridging organizations*, which, like *boundary spanners* provide mediation, but also emphasize knowledge coproduction and comanagement and adaptive governance of resources (Berkes, 2009; Crona & Parker, 2012). In our view, evidence bridges do not negotiate between parties or resolve conflicts; dictate or recommend a particular course of action; or act as conservation advocates.

As in medicine, a conservation evidence bridge should synthesize evidence *and* collaborate with practitioners to identify their needs and translate science accordingly. Segan et al. (2011) note regarding the medical equivalents, “In addition to the organizations that compile reviews of evidence (e.g., Clinical Evidence, Cochrane Library [https://www.cochrane.org/]), a second set of institutions in medicine… are responsible for synthesizing information for decision making. These institutions consider the effectiveness of a given intervention and other factors that might affect investment in the intervention, such as its cost, availability of alternative interventions to achieve the same objective, and effect on the patient and society of not acting.” It is this second set of institutions (or individuals) that is needed in conservation to supplement and complement the work of organizations that compile and review environmental evidence, such as Conservation Evidence (CE) (https://www.conservationevidence.com/) and Collaboration for Environmental Evidence (CEE) (https://www.environmentalevidence.org/). Organizations like CE and CEE partially fulfill the role of evidence bridges in conservation but by themselves cannot be considered fully formed evidence bridges. Evidence bridges may either synthesize evidence independently or use syntheses from other organizations, but their essential role is to convert syntheses into timely and useable information for practitioners. This process is an effective way to translate knowledge (Elueze, 2015; Lomas, 2007; Traynor et al., 2014) to promote uptake of evidence (Nguyen et al., 2017). This process encourages practitioners to make decisions openly and transparently and primary researchers to produce practice‐relevant (i.e., applied) evidence.

Medical evidence bridges produce need assessments and write evidence‐based clinical practice guidelines and summaries of empirical evidence. They develop and implement knowledge translation plans and strategic communications and dissemination plans to reach practitioners. Their overall mandate is to summarize, translate, and disseminate relevant evidence clearly and concisely to medical practitioners (Dicks et al., 2014; Haynes, 2001). The evidence is available in a quickly digestible form, minimizing the need for practitioners to hunt down evidence, thus increasing the likelihood that they will find and use it. Although medical practitioners may also struggle with the need for suitable evidence summaries (e.g., Bensing, 2000; Kahn et al., 2011; Scutchfield & Lamberth, 2010), we argue the gap in conservation is much greater.

## CONTRASTING EVIDENCE BRIDGES WITH ORGANIZATIONS THAT SYNTHESIZE CONSERVATION EVIDENCE

We contrasted evidence bridges with 2 existing organizations that compile and review conservation evidence. The CEE publishes systematic reviews, systematic maps, and meta‐analyses. These syntheses are generally either commissioned or driven by scientists. In contrast, an evidence bridge would synthesize research on topics selected through broad‐based consultation with a community of practitioners. In medicine, commissioned syntheses are the exception, and stakeholders help to define review questions. There is little evidence regarding the use of CEE by conservation practitioners. Few systematic reviews in conservation and environmental management contain conclusions that would be considered practical for on‐the‐ground management interventions (Cook et al., 2013b). In contrast, one of the primary medical intermediaries, Cochrane, has over 10,000 members and 31,000 contributors, including health care professionals and patients from over 130 countries (Cassels, 2013; Cochrane Library, 2020; von Elm et al., 2013).

Conservation Evidence maintains a growing database of “subject‐wide evidence syntheses” (i.e., searchable synopses [Sutherland & Wordley, 2018]) that has been integrated into several practitioner‐focused resources and decision‐support tools by “evidence champions” (Sutherland et al., 2019), such as the IUCN Red List, the National Biodiversity Network, and Conservation Management System software [CMSi]. However, as with CEE, CE does not select topics based on consultation with practitioners and currently lacks evidence of its effectiveness (Sutherland et al., 2019). At present, the only study documenting its use by practitioners indicates that it influenced choices of management in favor of more effective interventions (Walsh et al., 2015).

These conservation evidence organizations do an excellent job of synthesizing and summarizing information. But they only partially fulfill the role of evidence bridges in conservation (Appendix S6). They could broaden their roles as fully formed evidence bridges by increasing engagement with practitioners to source synthesis topics and delivering targeted synthesis products to practitioners. This would likely involve partnering with, hiring, or establishing dedicated knowledge brokers.

## ORGANIZATIONS THAT PARTIALLY FILL THE ROLE OF EVIDENCE BRIDGES

We found few organizations that might meet all the criteria (Appendix S6) for evidence bridges in conservation (Table 2). In this search, we reviewed literature on evidence intermediaries in conservation (Appendix S3). We also shared an anonymous questionnaire on Twitter in July–August 2020, which was seen 5780 times and had 193 interactions (Appendix S4). Some organizations synthesize evidence but do not generally source topics from practitioners or distribute syntheses through direct exchanges with practitioners (e.g., CEE and CE). Other organizations work with practitioners but do not produce practitioner‐focused evidence syntheses and tools (e.g., National Environmental Science Program Threatened Species Recovery Hub [https://www.nespthreatenedspecies.edu.au/]). Some evidence bridging work is contributing to conservation efforts, but it is unrecognized, often unplanned (Meyer, 2010), and only reaches a limited set of practitioners. Our review confirmed observations of other authors that the infrastructure that fully bridges evidence from researchers to practitioners still needs substantial development in conservation (Dicks et al., 2014; Farwig et al., 2017; Segan et al., 2011).

It was outside of the scope of this review to describe every organization in conservation and environmental management for its evidence‐bridging capacity and acknowledge that there is a growing recognition of the need and increasing in‐house capacity for synthesis work within government agencies. For example, Parks Canada (https://www.pc.gc.ca/en/index) (Government of Canada) recently established a *knowledge mobilization* group, which we identified through personal contacts and which we would likely not have been aware had we not been Canadian (personal communication). Nevertheless, based on our review, we conclude that actual evidence bridges are generally very difficult to find in conservation, suggesting that their number is currently insufficient to form a readily accessible broad‐based resource for conservation practitioners.

Despite this, we found 4 organizations that fulfill the criteria for evidence bridges (FRI Research [https://friresearch.ca/], Electric Power Research Institute [https://www.epri.com/], National Council for Air and Stream Improvement [NCASI] [https://www.ncasi.org/], and Rights‐of‐Way as Habitat Working Group [http://rightofway.erc.uic.edu/]) that provide examples of how evidence bridges can influence conservation practice. For example, NCASI identifies and prioritizes environmental issues related to forest products based on input from industry advisory panels. Individuals within NCASI synthesize forestry science results for practitioners in the forest‐product sector. Their successes in fostering use of evidence in practice, and the successes of the other organizations in Appendix S6, provide a model for evidence bridges in other sectors (Appendix S5).

## LIMITATIONS IN THE ROLES OF RESEARCHERS AND PRACTITIONERS

Neither primary researchers nor practitioners are well equipped to form evidence bridges themselves because of their different roles, skill sets, incentives, and constraints. Several authors argue that conservation researchers themselves should build evidence bridges to conservation practitioners (e.g., Arlettaz et al., 2010; Fabian et al., 2019; Laurance et al., 2012; Pullin & Knight, 2012; Rose, 2015) and even have an ethical responsibility to do so (Lacey et al., 2015). They argue that if researchers would communicate their work in user‐friendly ways, it would be more readily taken up and used by practitioners. Although we agree this can succeed in specific situations, it is not a general solution to the overall goal of improving the use of evidence in conservation decisions. Primary researchers are specialists in conducting research and publishing in the peer‐reviewed scientific literature. They generally lack strong links with practitioners who could use their work. Relying on primary researchers to build evidence bridges is problematic because the varying incentives provided by potential funding opportunities, citation rates, and media scores, such as Altmetric, make it difficult for individual researchers to provide independent and unbiased syntheses of the evidence (Martinson et al., 2009). We suggest that evidence bridges would help “relieve scientists from a task which they are not trained for and which they may be reluctant to do” (Farwig et al., 2017).

It is equally unrealistic to expect practitioners to form evidence bridges by seeking out and synthesizing the evidence needed for their particular problem. Conservation practitioners are highly diverse, ranging from individual landowners, to industrial employees, to government managers, with widely varying needs and skill sets. They are highly constrained by funding and especially by time (Cvitanovic et al., 2014; Nguyen et al., 2018; Pullin & Knight 2005; Walsh et al., 2015). Moreover, the conservation literature is vast. Few practitioners have the training or the time to distill the relevant and scientifically credible information they need. In many cases, practitioners feel overloaded by information, without the means to synthesize it into coherent evidence (e.g., Girling & Gibbs 2019; Safford et al., 2017). Nakagawa et al. (2020) stated, “it has been nearly impossible to keep up with the deluge of information made available to support…our daily decisions.” Evidence bridges would screen, evaluate, translate, and convey the existing science, thus allowing practitioners to use evidence rather than ignoring it because it is confusing (Table 1). Evidence bridges would also add value by facilitating interactions between practitioners and researchers.

## ROLE OF VALUES IN CONSERVATION DECISIONS

Conservation decisions usually involve multiple players with multiple competing objectives and values, which affect and may override the use of evidence, especially if it is hard to find. Values are, therefore, a fundamental component of conservation decision making, influencing conservation effectiveness (Buschke et al., 2019; Johnson et al., 2015; Toomey et al., 2017). Practitioners may also apply their own tacit knowledge, based on their intuition or experience, when making decisions (Table 1) (Hulme, 2014; Roux et al., 2006). Basing decisions exclusively on values or tacit knowledge can lead to conflict (examples in Redpath et al. [2013]) and biased and inappropriate decisions (Dicks et al., 2014). However, decisions that do not account for values may uphold “a perception of the disconnected ivory tower of science” (Rose, 2018).

Evidence bridges can help navigate this challenge because they engage with practitioners. As a result, they are aware of the values influencing decisions. A key benefit of evidence bridges, like *boundary organizations*, is then their capacity to cross the divide between evidence and values (Guston, 1999). This can also happen in some specific situations where practitioners and scientists work directly together. For example, scientists and land managers incorporated evidence and values to determine the most effective method for restoring habitat for flatwood salamanders (*Ambystoma cingulatum*) in Florida (O'Donnell et al., 2017). However, this is the exception in conservation decision making (Table 1). Evidence bridges would help build relationships between conservation practitioners and researchers systematically.

## MODEL FOR CREATING EVIDENCE BRIDGES IN CONSERVATION

In medicine, “where evidence‐based clinical practice is now routine” (Dicks et al., 2014), evidence bridges include not only government health departments but also independent regulatory bodies (e.g., Royal College of Physicians and Surgeons [https://www.royalcollege.ca/rcsite/home‐e], American College of Physicians [https://www.acponline.org]), nonprofit health organizations (e.g., American Health Association [https://americanhealthassoc.org/], Canadian Pediatric Society [https://www.cps.ca/]), and advocacy and specialty societies (e.g., American Cancer Society [https://www.cancer.org/], Alzheimer's Society [https://alzheimer.ca/en]). About 30% of evidence bridges in medicine are based in universities, about 10% in foundations or research funding agencies, and the remaining 60% are in the health system (Lomas, 2007).

Medicine is a much larger (and older) field than conservation and much more heavily funded. In medicine, some evidence bridges are charitable organizations (e.g., Cochrane), and some are funded by governments either within government agencies (e.g., Canadian Institutes of Health Research, Agency for Healthcare Research and Quality [https://www.ahrq.gov/]) or at arm's length (e.g., Centre for Effective Practice [https://cep.health/]). The latter organizations may also be partly privately funded. Some are housed at a university (e.g., Machealth [https://machealth.ca/]). In some cases, the practitioners using the evidence pay the evidence bridge for access via membership fees or dues (e.g., UpToDate [https://www.uptodate.com/home], Lexicomp [https://www.wolterskluwercdi.com/lexicomp‐online/]).

In principle, all these funding sources could also be used for evidence bridges in conservation. One funding source that creates particular challenges in medicine is industry funding, such as from pharmaceutical companies. This compromises objectivity in evidence synthesis and therefore the ability of the evidence bridge to perform its central role of providing the best, most up‐to‐date evidence to practitioners. In conservation, a similar challenge would arise if, for example, substantial funding for an evidence bridge were obtained from natural resource extraction industries. Thus, funding sources that compromise independence and transparency should be avoided or restricted.

Given the diversity of different issues faced in conservation, and the lack of dedicated funding, a single evidence‐bridging organization for all conservation decision making will not be practical. One approach to build consistency in evidence bridges would be to develop a college for conservation decision making modeled after the College of Physicians and Surgeons. Such a college could bring together and form a clearinghouse for conservation evidence bridges. There are several ways to develop such a college, potentially based on an existing organization. For example, the CEE could be a good starting point for such a professional community (Posner & Cvitanovic, 2019) of conservation evidence bridges. It already has a distributed set of centers (Lomas et al., 2007). A natural extension would be to extend its evidence synthesis activities to include evidence‐bridging activities. Each center could reach out to a broad segment of conservation practitioners, and the organizations with whom they network, to identify research topics most in need of evidence synthesis. Evidence bridges within the college could adopt synthesis standards developed by CEE, as well as communication and delivery standards. Certification could be used to ensure quality of evidence bridges in meeting practitioner needs in evidence synthesis (Farwig et al., 2017).

A college for conservation decision making could track which practitioners need evidence regarding which issues and which evidence bridges are synthesizing evidence for which broad issues. A college for conservation decision making could then direct practitioners to evidence bridges and direct evidence bridges to groups of practitioners in need of evidence. For example, a practitioner within the forest‐product sector could contact the evidence college and then be directed to an evidence bridge such as NCASI. The college would also serve to identify common evidence gaps across evidence bridges to avoid repetition and redundancy.

## CONCLUSION

We argue that full evidence bridges from research to practice are lacking and sorely needed in conservation. Such evidence bridges need to interact with practitioners to determine evidence needs and provide summarized, up‐to‐date, highly visible, and accessible evidence directly to practitioners for on‐the‐ground decision making. Several organizations help reduce the gap between evidence and practice and could serve as a platform for building additional components of evidence bridges in conservation. Evidence bridges require expertise in research and evidence synthesis but also expertise in communicating with conservation practitioners in need of evidence. We believe that professional societies, such as the Society for Conservation Biology (https://conbio.org/), Ecological Society of America (https://www.esa.org/), and British Ecological Society (https://www.britishecologicalsociety.org/), have an opportunity to play a leadership role in identifying and encouraging development of an appropriate model, such as a college for conservation decision making, to fill this gap.

## Supporting information


**Appendix S1**. A list of 18 example commentary papers on the “knowledge‐action” or “research‐implementation” gap.
**Appendix S2**. We systematically searched (May 2020) in Web of Science––Core Collection (309 records) and Scopus (452 records) for relevant articles using search terms listed below.
**Appendix S3**. We undertook an in‐depth review of the literature to find evidence intermediaries in the field of conservation and environmental management.
**Appendix S4**. Anonymous questionnaire distributed over Twitter July–August 2020.
**Table S5.1**. Bright spots: excellent examples of how evidence intermediary organizations have influenced conservation practice and what we can learn from these examples.
**Appendix S6**. A sample of existing organizations that partially fulfill the role of evidence bridges in conservation (ordered alphabetically).
