# Supplementary material for: Bridging research and practice in conservation
Source: Conserv Biol. 2021 Jun 4;35(6):1725–37. doi: 10.1111/cobi.13732 (PMC9291548; doi:10.1111/cobi.13732)
Supplement: Supplementary file 1 — Appendix S1. A list of 18 example commentary papers on the “knowledge‐action” or “research‐implementation” gap. Appendix S2. We systematically searched (May 2020) in Web of Science––Core Collection (309 records) and Scopus (452 records) for relevant articles using search terms listed below. Appendix S3. We undertook an in‐depth review of the literature to find evidence intermediaries in the field of conservation and environmental management. Appendix S4. Anonymous questionnaire distributed over Twitter July–August 2020. Table S5.1. Bright spots: excellent examples of how evidence intermediary organizations have influenced conservation practice and what we can learn from these examples. Appendix S6. A sample of existing organizations that partially fulfill the role of evidence bridges in conservation (ordered alphabetically). [file COBI-35-1725-s001.docx]

***Conservation Biology***

Electronic Suppporting Information

Title: **Bridging Research and Practice in Conservation**

<https://doi.org/10.1111/cobi.13732>

**Authors:** Andrew N. Kadykalo, Rachel T. Buxton, Peter Morrison, Christine M. Anderson, Holly Bickerton, Charles M. Francis, Adam C. Smith, Lenore Fahrig

**Appendix S1.** A list of 18 example commentary papers on the ‘knowledge-action’ or ‘research-implementation’ gap. See also *Biological Conservation*’s Special Issue on “Implementation Spaces in Conservation Science”: <https://www.sciencedirect.com/journal/biological-conservation/special-issue/109PW5Z450C>

2007. The great divide. Nature **450**(7167):135-136. DOI: 10.1038/450135b.

Arlettaz R, Schaub M, Fournier J, Reichlin TS, Sierro A, Watson JEM, Braunisch V. 2010. From

publications to public actions: When conservation biologists bridge the gap between research and implementation. BioScience **60**(10):835-842. DOI: 10.1525/bio.2010.60.10.10.

Bertuol-Garcia D, Morsello C, C NE-H, Pardini R. 2018. A conceptual framework for

understanding the perspectives on the causes of the science-practice gap in ecology and conservation. Biological reviews of the Cambridge Philosophical Society **93**(2):1032-1055. DOI: 10.1111/brv.12385:10.1111/brv.12385.

Cook CN, Mascia MB, Schwartz MW, Possingham HP, Fuller RA. 2013. Achieving

conservation science that bridges the knowledge-action boundary. Conservation Biology **27**(4):669-678. DOI: 10.1111/cobi.12050.

Cowling R. 2005. Maintaining the research-implementation continuum in conservation. Society

for Conservation Biology Newsletter **12**(1-19).

Dubois NS, Gomez A, Carlson S, Russell D. 2019. Bridging the research‐implementation gap

requires engagement from practitioners. Conservation Science and Practice **2**(1): e134. DOI: 10.1111/csp2.134.

Habel JC, Gossner MM, Meyer ST, Eggermont H, Lens L, Dengler J, Weisser WW. 2013. Mind

the gaps when using science to address conservation concerns. Biodiversity and Conservation **22**(10):2413-2427. DOI: 10.1007/s10531-013-0536-y.

Hulme PE. 2014. Editorial: Bridging the knowing-doing gap: Know-who, know-what, know-

why, know-how and know-when. Journal of Applied Ecology **51**(5):1131-1136. DOI: 10.1111/1365-2664.12321.

Jarvis RM, Borrelle SB, Bollard Breen B, Towns DR. 2015. Conservation, mismatch and the

research-implementation gap. Pacific Conservation Biology **21**:105-107. DOI: 10.1071/PC14912.

Jarvis RM, Borrelle SB, Forsdick NJ, Pérez-Hämmerle K-V, Dubois NS, Griffin SR, Recalde-

Salas A, Buschke F, Rose DC, Archibald CL, Gallo JA, Mair L, Kadykalo AN, Shanahan D, Prohaska BK. 2020. Navigating spaces between conservation research and practice: Are we making progress? Ecological Solutions and Evidence **1**:e12028. DOI: 10.1002/2688-8319.12028.

Knight AT, Cowling RM, Rouget M, Balmford A, Lombard AT, Campbell BM. 2008.

Knowing but not doing: Selecting priority conservation areas and the research implementation gap. Conservation Biology **22**(3):610-617. DOI: 10.1111/j.1523-1739.2008.00914.x.

Maas B, Toomey A, Loyola, R., 2019. Exploring and expanding the spaces between research and

implementation in conservation science. Biological Conservation **240**:108290. DOI: 10.1016/j.biocon.2019.108290

Mihók B, Kovács E, Balázs B, Pataki G, Ambrus A, Bartha D, Czirák Z, Csányi S, Csépányi P,

Csőszi M, et al. 2015. Bridging the research-practice gap: Conservation research priorities in a Central and Eastern European country. Journal for Nature Conservation **28**:133-148. DOI: 10.1016/j.jnc.2015.09.010.

Roux DJ, Rogers KH, Biggs HC, Ashton PJ, Sergeant A. 2006. Bridging the science-

management divide: Moving from unidirectional knowledge transfer to knowledge interfacing and sharing. Ecology and Society **11**(1):4. DOI: 10.5751/ES-01643-110104

Sunderland T, Sunderland-Groves J, Shanley P, Campbell B. 2009. Bridging the gap: How can

information access and exchange between conservation biologists and field practitioners be improved for better conservation outcomes? Biotropica **41**(5):549-554. DOI: 10.1111/j.1744-7429.2009.00557.x.

Stern MJ, Briske DD, Meadow AM. 2021. Opening learning spaces to create actionable

knowledge for conservation. Conservation Science and Practice e378. DOI: 10.1111/csp2.378

Toomey AH, Knight AT, Barlow J. 2017. Navigating the space between research and

implementation in conservation. Conservation Letters **10**(5):619-625. DOI: 10.1111/conl.12315.

Walsh JC, Dicks LV, Raymond CM, Sutherland WJ. 2019. A typology of barriers and enablers

of scientific evidence use in conservation practice. Journal of Environmental Management **250**(15):109481**.** DOI: 10.1016/j.jenvman.2019.109481.

**Appendix S2**. We systematically searched (May 2020) in Web of Science – Core Collection (309 records) and Scopus (452 records) for relevant articles using search terms listed below. Each record was screened based on its title and/or abstract for relevance. We specifically sought studies that surveyed conservation practitioners, using interviews or questionnaires, to determine how they make decisions. We further reviewed the bibliographies of all selected articles for any additional relevant studies that might have been missed in the initial search.

Search terms and phrases used:

((TITLE: “evidence” OR “knowledge” OR “information” OR “scien*” OR “literature”) AND (TITLE: "use" OR "used" OR "using" OR "utility" OR "gather*" OR "role" OR "base*" OR "consider*" OR “exchange” OR “mobilization” OR “mobilisation”) AND (TITLE-ABS-KEY: “manager*” OR “farmer*” OR “land*owner*” OR “industry” OR “industries” OR “commercial” OR “stakeholder*” OR “rancher*” OR “resident*” OR “household*” OR “ENGO*” OR “NGO*” OR “proponent*” OR “citizen*” OR “practitioner*” OR “knowledge user*” OR “end-user*”) AND (TITLE: “conservation” OR “biodiversity” OR “ecology” OR (“environment*” AND “manag*”) OR (“natural resource*” AND “manag*”) OR (“environment*” AND “practice”) OR (“environment*” AND “science*”)))

**Appendix S3**. We undertook an in-depth review of the literature to find evidence intermediaries in the field of conservation and environmental management. We found 44 papers with relevant information.

We systematically searched (July 2020) in Web of Science – Core Collection (515 records) and Scopus (651 records) for literature describing evidence intermediaries in conservation and environmental management using search terms listed below. We screened all papers for relevance based on their title and/or abstract. We further reviewed the bibliographies of relevant articles. See Appendix C for the list of 44 reviewed records.

Search terms and phrases used:

((TITLE-ABS-KEY: “boundary scien*” OR “boundary org*” OR “boundary span*” OR “bridging agent*” OR “bridging org*” OR “bridging individual*” OR “evidence broker*” OR “knowledge broker*” OR “intermediar*”) AND (TITLE-ABS-KEY: conservation OR "environmental org*" OR "environmental governance" OR "environmental sector" OR "environmental research" OR "environmental practice" OR "environmental manage*" OR "environmental scien*" OR "environmental decision*" OR "natural resource manage*"))

The list of eligible (included) records on evidence intermediaries in conservation and environmental management that we reviewed in detail.

Bednarek AT, Shouse B, Hudson CG, Goldburg R. 2016. Science-policy intermediaries from a

practitioner’s perspective: The Lenfest Ocean Program experience. Science and Public Policy **43**(2):291-300. DOI: 10.1093/scipol/scv008.

Bednarek AT, Wyborn C, Cvitanovic C, Meyer R, Colvin RM, Addison PFE, Close SL, Curran

K, Farooque M, Goldman E, Hart D, et al. 2018. Boundary spanning at the science-policy interface: The practitioners' perspectives. Sustainability Science **13**(4):1175-1183. DOI: 10.1007/s11625-018-0550-9.

Beratan KK. 2019. Improving problem definition and project planning in complex natural

resource management problem situations using knowledge brokers and visual design principles. Ecology and Society:**24**(2):31. DOI: 10.5751/ES-10815-240231

Berkes F. 2009. Evolution of co-management: Role of knowledge generation, bridging

organizations and social learning. Journal of Environmental Management **90**(5):1692-1702. DOI: 10.1016/j.jenvman.2008.12.001.

Brownson K, Chappell J, Meador J, Bloodgood J, Howard J, Kosen L, Burnett H, Gancos-

Crawford T, Guinessey E, Heynen N, et al. 2020. Land trusts as conservation boundary organizations in rapidly exurbanizing landscapes: A case study from Southern Appalachia. Society & Natural Resources **33**(10):1309-1320. DOI: 10.1080/08941920.2020.1731034.

Carr A, Wilkinson R. 2005. Beyond participation: Boundary organizations as a new space for

farmers and scientists to interact. Society & Natural Resources **18**(3):255-265. DOI: 10.1080/08941920590908123.

Cash DW. 2001. “In order to aid in diffusing useful and practical information”: Agricultural

extension and boundary organizations. Science, Technology, & Human Values **26**(4):431-453. DOI: 10.1177/016224390102600403.

Cash DW, Clark WC, Alcock F, Dickson NM, Eckley N, Guston DH, Jager J, Mitchell RB.

2003. Knowledge systems for sustainable development. Proceedings of the National Academy of Sciences **100**(14):8086-8091. DOI: 10.1073/pnas.1231332100.

Cash DW, Borck JC, Patt AG. 2006. Countering the loading-dock approach to linking

science and decision making. Science, Technology, & Human Values **31**(4):465-494. DOI: 10.1177/0162243906287547.

Cook CN, Mascia MB, Schwartz MW, Possingham HP, Fuller RA. 2013. Achieving

conservation science that bridges the knowledge-action boundary. Conservation Biology **27**(4):669-678. DOI: 10.1111/cobi.12050.

Crona BI, Parker JN. 2012. Learning in support of governance: Theories, methods, and a

framework to assess how bridging organizations contribute to adaptive resource governance. Ecology and Society **17**(1):32. DOI: 10.5751/es-04534-170132.

Cvitanovic C, Hobday AJ, van Kerkhoff L, Wilson SK, Dobbs K, Marshall NA. 2015.

Improving knowledge exchange among scientists and decision-makers to facilitate the adaptive governance of marine resources: A review of knowledge and research needs. Ocean & Coastal Management **112**:25-35. DOI: 10.1016/j.ocecoaman.2015.05.002.

Cvitanovic C, McDonald J, Hobday AJ. 2016. From science to action: Principles for

undertaking environmental research that enables knowledge exchange and evidence-based decision-making. Journal of Environmental Management **183**(3):864-874. DOI: 10.1016/j.jenvman.2016.09.038.

Cvitanovic C, Cunningham R, Dowd AM, Howden SM, van Putten EI. 2017. Using

social network analysis to monitor and assess the effectiveness of knowledge brokers at connecting scientists and decision-makers: An Australian case study. Environmental Policy and Governance **27**(3):256-269. DOI: 10.1002/eet.1752.

Cvitanovic C, Lof MF, Norstrom AV, Reed MS. 2018. Building university-based

boundary organisations that facilitate impacts on environmental policy and practice. PLoS One **13**(9):e0203752. DOI: 10.1371/journal.pone.0203752.

Dicks LV, Walsh JC, Sutherland WJ. 2014. Organising evidence for environmental

management decisions: A '4s' hierarchy. Trends in Ecology & Evolution **29**(11):607-613. DOI: 10.1016/j.tree.2014.09.004.

Duncan R, Robson-Williams M, Edwards S. 2020. A close examination of the role and

needed expertise of brokers in bridging and building science policy boundaries in environmental decision making. Palgrave Communications **6**(1):64. DOI: 10.1057/s41599-020-0448-x.

Eanes FR, Singh AS, Bulla BR, Ranjan P, Fales M, Wickerham B, Doran PJ, Prokopy LS.

2019. Crop advisers as conservation intermediaries: Perceptions and policy implications for relying on nontraditional partners to increase U.S. Farmers’ adoption of soil and water conservation practices. Land Use Policy **81**:360-370. DOI: 10.1016/j.landusepol.2018.10.054.

Farwig N, Ammer C, Annighöfer P, Baur B, Behringer D, Diekötter T, Hotes S, Leyer I, Müller

J, Peter F, et al. 2017. Bridging science and practice in conservation: Deficits and challenges from a research perspective. Basic and Applied Ecology **24**:1-8. DOI: 10.1016/j.baae.2017.08.007.

Fazey I, Evely AC, Reed MS, Stringer LC, Kruijsen J, White PCL, Newsham A, Jin L, Cortazzi

M, Phillipson J, et al. 2012. Knowledge exchange: A review and research agenda for environmental management. Environmental Conservation **40**(1):19-36. DOI: 10.1017/s037689291200029x.

Fazey I, Bunse L, Msika J, Pinke M, Preedy K, Evely AC, Lambert E, Hastings E, Morris S,

Reed MS. 2014. Evaluating knowledge exchange in interdisciplinary and multi-stakeholder research. Global Environmental Change **25**:204-220. DOI: 10.1016/j.gloenvcha.2013.12.012.

Gustafsson KM, Lidskog R. 2018. Boundary organizations and environmental governance:

Performance, institutional design, and conceptual development. Climate Risk Management **19**:1-11. DOI: 10.1016/j.crm.2017.11.001.

Guston DH. 1999. Stabilizing the boundary between us politics and science: The role of the

office of technology transfer as a boundary organization. Social Studies of Science **29**(1):87-111. DOI: 10.1177/030631299029001004.

Guston DH. 2001. Boundary organizations in environmental policy and science: An

introduction. Science, Technology, & Human Values **26**(4):399-408. DOI: 10.1177/016224390102600401.

Hulme PE. 2014. Editorial: Bridging the knowing-Doing gap: Know-who, know-what, know-

why, know-how and know-when. Journal of Applied Ecology **51**(5):1131-1136. DOI: 10.1111/1365-2664.12321.

Jensen-Ryan DK, German LA. 2019. Environmental science and policy: A meta-synthesis

of case studies on boundary organizations and spanning processes. Science and Public Policy **46**(1):13-27. DOI: 10.1093/scipol/scy032.

Lacey J, Howden M, Cvitanovic C, Colvin RM. 2018. Understanding and managing trust at the

climate science–policy interface. Nature Climate Change **8**(1):22-28. DOI: 10.1038/s41558-017-0010-z.

Lidskog R. 2014. Representing and regulating nature: Boundary organisations, portable

representations, and the science–policy interface. Environmental Politics **23**(4):670-687. DOI: 10.1080/09644016.2013.898820.

Meyer M. 2010. The rise of the knowledge broker. Science Communication **32**(1):118-127.

DOI: 10.1177/1075547009359797.

Michaels S. 2009. Matching knowledge brokering strategies to environmental policy problems

and settings. Environmental Science & Policy **12**(7):994-1011. DOI: 10.1016/j.envsci.2009.05.002.

Nguyen VM, Young N, Cooke SJ. 2017. A roadmap for knowledge exchange and

mobilization research in conservation and natural resource management. Conservation Biology **31(**4):789-798. DOI: 10.1111/cobi.12857.

Parker J, Crona B. 2012. On being all things to all people: Boundary organizations and the

contemporary research university. Social Studies of Science **42**(2):262-289. DOI: 10.1177/0306312711435833.

Pielke JRA. 2007. The Honest Broker: Making Sense of Science in Policy and Politics.

Cambridge University Press, Cambridge.

Pietri D, McAfee S, Mace A, Knight E, Rogers L, Chornesky E. 2011. Using science to

inform controversial issues: A case study from the California Ocean Science Trust. Coastal Management **39**(3):296-316. DOI: 10.1080/08920753.2011.566118.

Posner SM, Cvitanovic C. 2019. Evaluating the impacts of boundary-spanning activities at

the interface of environmental science and policy: A review of progress and future research needs. Environmental Science & Policy **92**:141-151. DOI: 10.1016/j.envsci.2018.11.006.

Reed MS, Stringer LC, Fazey I, Evely AC, Kruijsen JHJ. 2014. Five principles for

the practice of knowledge exchange in environmental management. Journal of Environmental Management **146**:337-345. DOI: 10.1016/j.jenvman.2014.07.021.

Rose DC. 2015. The case for policy-relevant conservation science. Conservation Biology

**29**(3):748-754. DOI: 10.1111/cobi.12444.

Roux DJ, Rogers KH, Biggs HC, Ashton PJ, Sergeant A. 2006. Bridging the science-

management divide: Moving from unidirectional knowledge transfer to knowledge interfacing and sharing. Ecology and Society **11**(1):4. DOI: 10.5751/ES-01643-110104.

Roux DJ, Nel JL, Cundill G, O’Farrell P, Fabricius C. 2017. Transdisciplinary research

for systemic change: Who to learn with, what to learn about and how to learn. Sustainability Science **12**(5):711-726. DOI: 10.1007/s11625-017-0446-0.

Roux DJ, Kingsford RT, Cook CN, Carruthers J, Dickson K, Hockings M. 2019. The

case for embedding researchers in conservation agencies. Conservation Biology **33**(6):1266-1274. DOI: 10.1111/cobi.13324.

Safford HD, Sawyer SC, Kocher SD, Hiers JK, Cross M. 2017. Linking knowledge to

action: The role of boundary spanners in translating ecology. Frontiers in Ecology and the Environment **15**(10):560-568. DOI: 10.1002/fee.1731.

Shaw J, Danese C, Stocker L. 2013. Spanning the boundary between climate science and

coastal communities: Opportunities and challenges. Ocean & Coastal Management **86**:80-87. DOI: 10.1016/j.ocecoaman.2012.11.008.

Stewart J, Tyler ME. 2019. Bridging organizations and strategic bridging functions in

environmental governance and management. International Journal of Water Resources Development **35**(1):71-94. DOI: 10.1080/07900627.2017.1389697

Turner II BL, Esler KJ, Bridgewater P, Tewksbury J, Sitas N, Abrahams B, Chapin FS,

Chowdhury RR, Christie P, Díaz S, et al. 2016. Socio-environmental systems (SES) research: What have we learned and how can we use this information in future research programs. Current Opinion in Environmental Sustainability **19**:160-168. DOI: 10.1016/j.cosust.2016.04.001.

**Appendix S4.** Anonymous questionnaire distributed over Twitter July-August 2020. Response data are provided in Table 3 and Appendix E. The tweet was shared by several prominent organizations in the conservation community, e.g.,: Society for Conservation Biology, Society for Conservation Biology Social Science Working Group, Liber Ero Fellows, Young Ecosystem Services Specialists, etc.

*Tweet*


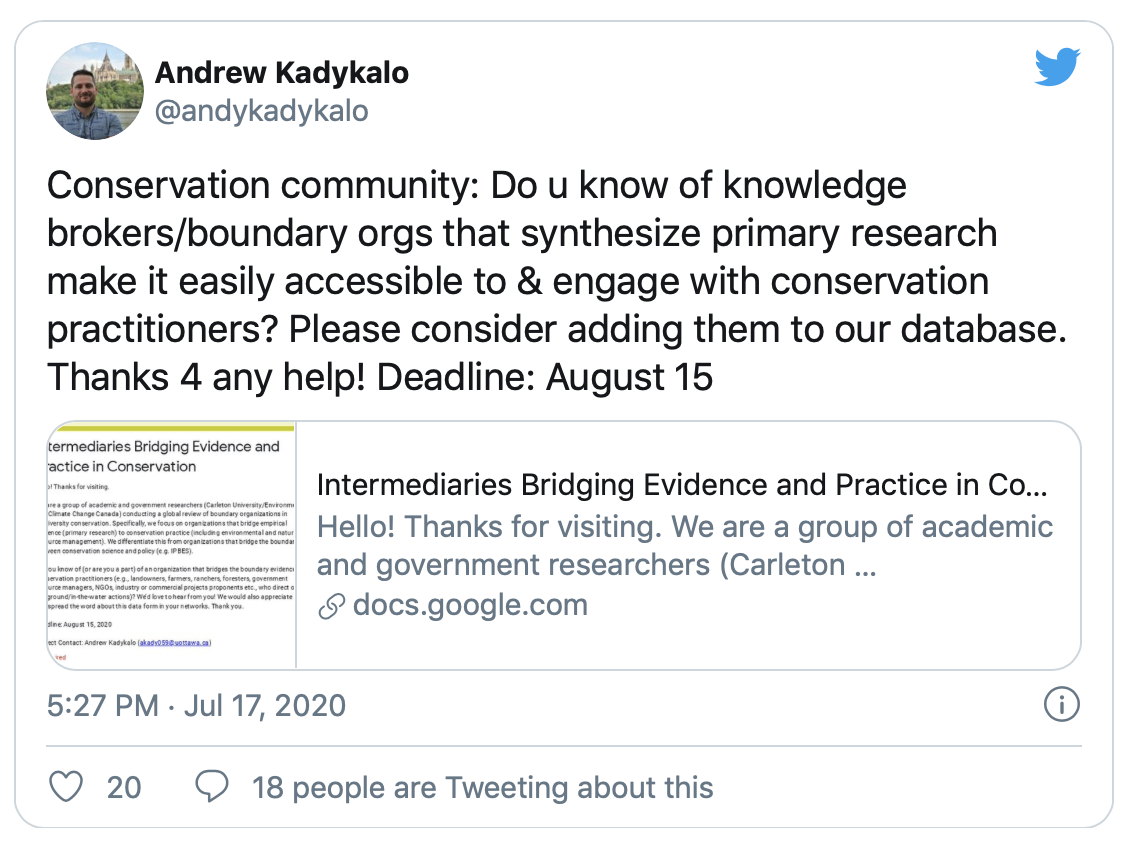


*Questionnaire Form*

Intermediaries Bridging Evidence and Practice in Conservation

Hello! Thanks for visiting.

We are a group of academic and government researchers (Carleton University/Environment and Climate Change Canada) looking for evidence intermediary organizations in biodiversity conservation. Specifically, we focus on organizations that bridge empirical evidence (primary research) to conservation practice (including environmental and natural resource management). We differentiate this from organizations that bridge the boundary between conservation science and policy (e.g. IPBES).

Do you know of (or are you a part) of an organization that bridges the boundary evidence to conservation practitioners (e.g., landowners, farmers, ranchers, foresters, government resource managers, NGOs, industry or commercial projects proponents etc., who direct on-the-ground/in-the-water actions)? We’d love to hear from you! We would also appreciate if you spread the word about this data form in your networks. Thank you.

Deadline: August 15, 2020

Project Contact: Andrew Kadykalo ([akady059@uottawa.ca](mailto:akady059@uottawa.ca))

* Required

Name of organization *

Your answer

Website for organization (org.)

Your answer

Type of conservation practitioners served (e.g., landowners, farmers, ranchers, foresters, government resource managers, NGOs, industry or commercial projects proponents etc., who direct on-the-ground/in-the-water actions) *

Your answer

What methods are used to deliver evidence-based information to those practitioners (e.g. journals, synopses, videos, workshops, briefing notes, fact sheets, etc.)?

Your answer

Location served: which region, country/countries, region, or are they international?

Your answer

Age: When was the org. founded?

Your answer

Size: How big is the org., measured by numbers of projects, numbers of staff, or annual revenues?

Your answer

Business Model: How does the org. cover their costs? Fee-for-service? Grants?

Your answer

Thematic Scope: What are their areas of expertise? How do they choose what to focus on? (geographically focused? topically focused?)

Your answer

Degree of Independence: Where is the org. housed? What is its affiliation with industry?

Your answer

Bright Spots: What are good examples of how this org. has influenced practice? What can we learn from these examples?

Your answer

Thank you for your participation.

**Appendix S5**

**Table S5.1.** Bright spots: excellent examples of how evidence intermediary organizations have influenced conservation practice and what we can learn from these examples.

| **Name of Organization** | **Bright Spots** |
| --- | --- |
| [Collaboration for Environmental Evidence](http://www.environmentalevidence.org/) | CEE has established the guidance and standards for evidence synthesis in the environmental sector and provides free materials to support conduct of syntheses and a free evidence service for decision makers. |
| [Conservation Evidence](http://www.conservationevidence.com/) | Influenced design of EU Agriculture Policy through demonstration that habitat and landscape features benefit biodiversity more than different crop types. |
| [Foundations of Success, Inc.](https://fosonline.org/) | A selection of bright spots based on FOS work:    - The Conservation Measures Partnership (CMP) and the spread of the Conservation Standards around the world - FOS and the founding members of CMP helped catalyze this movement, and the Conservation Coaches Network (CCNet) has been an important player in its spread. There is a true community of practice around using good conservation practices.  - Multiple smaller projects who reflect on their work and adapt their strategies using evidence on a monthly, bi-annual, and/or annual basis.  - Conservation Planning and courses at UW-Madison (part of the core curriculum for the Environmental Conservation M.S.) - students coming out of this program continue to appreciate the course and use what they've learned to work for conservation organizations and beyond  - Development of CMP working groups on evidence.  - USAID adopting evidence-based practice - through the Measuring Impact and Measuring Impact II projects, FOS and its partners have helped to instill adaptive management and evidence-based practices into conservation and integrated conservation programming. |
| [Foothills Research Institute](http://friresearch.ca/)  [fRI Research](http://friresearch.ca/) | One highlight is the success of the long-standing Grizzly Bear Program, and its work in identifying population of grizzly bears in Alberta. This led to provincial protection of the species. |
| [Great Lakes Acoustic Telemetry Observation System](https://glatos.glos.us/)  [(GLATOS)](https://glatos.glos.us/) | Understanding cross-jurisdictional movement of native exploited stocks as well as invasive species (ie grass carp and sea lamprey). GLATOS has also uncovered previously unknown movement patterns of commercially and recreationally important species. |
| [Great Lakes Fishery Commission](http://www.glfc.org/index.php) | Joint Strategic Plan for Great Lakes Fishery Management – see http://www.glfc.org/history.php. Also, the conduct of the binational sea lamprey control program is guided by the Commission and its research. |
| [Miistakis Institute](http://rockies.ca/) | Miistakis research has been used in work for highway wildlife crossing mitigation work; with sharing conservation science information with the public and decision-makers; and bringing greater awareness and support in land-use and recreation planning for the where/why we need to secure habitat, movement corridors and large landscape connectivity for wildlife; In working with the ranching community, practitioners are able to provide scientific backing to implement change with a conservation focus through Miistakis' accessible material; The Miistakis review of the Dead Man's Flats wildlife underpass within the 2012 Highway 1 report was used to argue against development at the underpass' entrance at the Municipal Governance Board. Although the pro-development contingent won that decision, the Miistakis review was pivotal in persuading the Government of Alberta to complete the land swap that will keep that underpass at least reasonably functional; The information provided by Miistakis helped craft process recommendations and a toolkit to integrate riparian protection into municipal land-use and water planning processes. These recommendations have been adopted and implemented and have helped conserve riparian areas; Miistakis's Municipal Flood and Drought Action Planning Primer - has been useful to work in central Alberta working with municipalities on issues related to water security/municipal planning Presentations/forums on citizen science have been helpful for work to understand the value add of citizen science for data collection and citizen engagement. |
| [National Environmental Science Program Threatened Species Recovery Hub](http://nespthreatenedspecies.edu.au/) | This organisation has generated a very wide range of research collaborations leading to practice change, including tools and guidelines used by land managers (the wide collaboration leading to the update of Australia's Threatened Plant Translocation Guidelines which informs translocation practice across the continent, the production of monitoring protocols and best practice and generation of a national conversation on threatened species monitoring in the book "Monitoring Threatened Species and Ecological Communities", promotion of science around and support social licence for feral predator control (especially cats), biodiversity in productive and urban landscapes, support for high-profile (and less high-profile) conservation programs across the country, research to support post-bushfire recovery in the wake of 2020 fires. https://www.nespthreatenedspecies.edu.au/publications-tools/ has many magazine articles, tools, guidelines, findings fact sheets etc that speak to the scope of the work. |
| [NatureServe Canada](http://www.natureserve.ca/) | NatureServe Network focuses on standard methodology for species assessments/mapping that are used by 90% of provincial, territorial and state governments in Canada and USA (enabling research, analysis across borders). Strong focus on Open Data. In the process of launching upgraded NatureServe Explorer platform to make NatureServe Network spatial and element data available to public at no cost. https://explorer.natureserve.org/ Canadian data precision will increase in coming months (first launch of spatial data is Aug 26 and will be 343 square miles precision---by end of fiscal CDN data available at up to 1 square mile, except for "sensitive species". |
| [Northern Australia Environmental Resources Hub](http://nespnorthern.edu.au/) | 1. Invasive para grass (Brachiaria mutica) threatens important cultural and ecological values of Nardab floodplains in Kakadu National Park. NESP researchers have built on long-term collaborations with Bininj/Mungguy Traditional Owners to develop and apply Bininj/Mungguy indicators of cultural-ecosystem health for the floodplains. These indicators are being used to identify priority areas for targeted para grass control and monitor the effectiveness of treatments.  2. Invasive gamba grass is transforming Australia’s northern savannas, replacing native species with dense stands of highly flammable grass that burns up to eight times the intensity of native grasses. NESP research is helping the rangers at Mary River National Park in the Northern Territory turn this invasion around, by guiding and providing evidence of the effectiveness of changes in their management activities to reduce fire frequency and carefully target spray treatments.  3. Gouldian finches live in small and mobile groups, and are difficult to find using standard field survey methods. NESP researchers have developed an eDNA test for the Gouldian finch (Erythrura gouldiae); the first for an endangered bird species. Gouldian finches live in small and mobile groups, and are difficult to find using standard survey methods. The test allows the finches to be detected from water samples collected from the small pools where they drink.  4. Feral pigs are a threat to nesting marine turtles, which are culturally important species for the Southern Wik Traditional Owners on Cape York. Collaborative research between the Cape York Indigenous organisation, Aak Puul Ngantam (APN) and the CSIRO is enabling Traditional Owners to more effectively manage feral pigs on their homelands. Using an app to collect data about turtle nest predation and success, and real time data visualisation about predation threats, APN Rangers can focus predator control on turtle habitat around peak nesting and hatching seasons. The APN data on feral pigs is also helping to manage biosecurity risks by informing surveillance activities under the Australian Government’s Northern Australian Quarantine Strategy. |
| [Pepperwood](https://www.pepperwoodpreserve.org/) | As a backbone organization for scientific collaborations to advance climate research (TBC3) and science/practitioner efforts for multi-jurisdiction, regional climate-wise connectivity (M2B) |
| [Rights-of-Way as Habitat Working Group](http://rightofway.erc.uic.edu/) | Over the past two years, the Rights-of-Way as Habitat Working Group led the development of the first nationwide Candidate Conservation Agreement with Assurances for the monarch butterfly. This project is a feat of collaboration among energy and transportation industry partners across the U.S. and the U.S. Fish and Wildlife Service in the name of conservation. |
| [Stockholm University Baltic Sea Centre (formerly the Baltic Eye project)](https://www.su.se/ostersjocentrum/english/) | See for example Cvitanovic et al. (2018)  Being part of the University has been impactful, giving the organization credibility and making people willing to listen. It has not been difficult at all to get access to decision-makers at Swedish or EU level, or practitioners, to discuss Baltic Sea issues, what can be done, to present the organizations communication material etc. |
| [The Society for Ecological Restoration](https://www.ser.org/) | In 2019, updated International Standards for the Practice of Ecological Restoration. This document is freely available from the organizational website and is used as the basis for restoration planning for projects all over the world. The organization is a partner for the UN Decade on Ecosystem Restoration, regularly work with the Convention on Biological Diversity, and the UN Convention to Combat Desertification. |
| [The Western Australian Biodiversity Science Institute](https://wabsi.org.au/) | Completion Criteria Framework, endorsed by government agency and adopted by industry: https://wabsi.org.au/our-work/projects/completion-criteria-and-risk-based-monitoring/ |
| [Yellowstone to Yukon Conservation Initiative](https://y2y.net/) | A few examples: broadest work and huge progress in protected areas and linkages over 25 years: https://y2y.net/work/impact; many individual stories on the blog: https://y2y.net/resources/news/; led the IUCN global guidelines for connectivity: https://y2y.net/blog/newly-published-global-guidelines-for-connectivity-conservation; synthesizing and advocating for stronger science in impact assessment: https://y2y.net/blog/strong-foundations-science-and-impact-assessment-in-canada; working with private landowners to secure critical linkage areas for wildlife movement: https://y2y.net/blog/vital-ground-and-yellowstone-to-yukon-protect-key-grizzly-habitat-along-kootenai-river-in-northwest-montana/ and https://thenelsondaily.com/news/nature-conservancy-canada-allows-frogs-and-bears-feel-safe-creston-valley-21074 |

**Appendix S6.** A sample of existing organizations that partially fulfill the role of evidence bridges in conservation (ordered alphabetically). Founded = year when the organization was founded. Business model = how the organization covers its costs. Thematic scope = area of expertise, whether geographically or topically focused. Associations = where the organization is housed and its degree of independence or affiliation with industry.


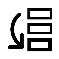
 Identifies research topics sourced from the priorities of practitioners


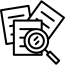
 Produces evidence syntheses that are directly useable by practitioners during on-the-ground decision-making


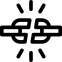
 Produces evidence syntheses that are primarily non-commissioned, avoiding or restricting economic or other interests that may affect objectivity and independence


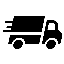
 Distributes easy-to-find and easy-to-use evidence syntheses to practitioners


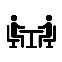
 Distributes evidence syntheses through face-to-face exchanges


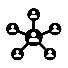
 Develops and maintains networks of connections among and between researchers and practitioners

| **Name of Organization** | **Type of practitioners served** | **Methods of delivering evidence-based information to those practitioners** | **Location served** | **Founded** | **Size** | **Business Model** | **Thematic Scope** | **Associations** | **Bridging criteria present (black) or absent (light grey)** |
| --- | --- | --- | --- | --- | --- | --- | --- | --- | --- |
| [Applied Ecology Resources (AER)](http://www.appliedecologyresources.org/) | Anyone wanting to search for information on applied ecology and the management of environmental resources | Peer-reviewed journal, research summaries, grey literature indexing and archiving, teaching resources, webinars, Evidence in Conservation Teaching Initiative (open access materials to aid teaching the core skills of evidence-based conservation) | Global/  International | 2020 | AER is still in development, but currently has 1.5 FTE in-house staff, plus 3 P/T Editors and is supported by an Advisory Board of 11 people | Income sources to enable long-term sustainability include membership fees at the organisation level, and gold open access journal | Topic focused: applied ecology, environmental and natural resource management, conservation | Applied Ecology Resources is run by the British Ecological Society, nonprofit organization | 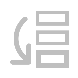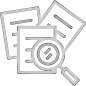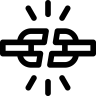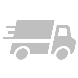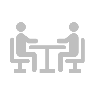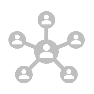 |
| [California Ocean Science Trust](https://www.oceansciencetrust.org/) | Primarily state government agencies (resource managers and policy-makers) and legislators | Formal science advisor to California's cabinet level agency overseeing coastal and ocean issues; convene expert panels of scientists to deliver science analyses, syntheses, and recommendations; convene joint science - manager workshops; perform peer reviews; deliver briefings to executive and legislative branch in state government. | California, U.S. | 2000 | Staff of 10 science-policy experts | Blended model of public and private grants and contracts, fee-for-service | Coast and ocean issues of priority to California state decision-makers | Independent 501(c)(3) nonprofit organization | 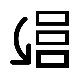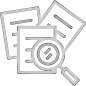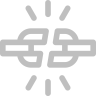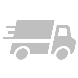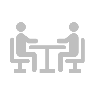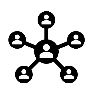 |
| [Collaboration for Environmental Evidence (CEE)](http://www.environmentalevidence.org/) | All practitioners | Systematic reviews and (evidence) maps; *Environmental Evidence* peer-reviewed journal; International Conference of the Collaboration for Environmental Evidence; RepOrting standards for Systematic Evidence Syntheses (ROSES); CEE Database of Evidence Reviews (CEEDER); workshops; social media | Global/  International | 2008 | Eight CEE Centres | Charity reliant on grants and donations | Global environmental management | Independent nonprofit | 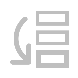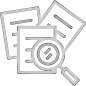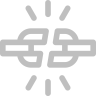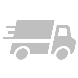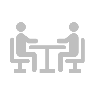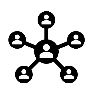 |
| [Conservation Evidence (CE)](http://www.conservationevidence.com/) | All practitioners | *Conservation Evidence* peer-reviewed journal; reports from conservation organizations; Searchable online database of conservation interventions synopses (summary paragraphs, key messages, expert assessments) and evidence assessments (Delphi-derived with multiple expert panels) plus 'What Works in Conservation' book, all open access | Global/  International | 2004 | 11 staff | Charitable grants and research grants | Biodiversity conservation, all taxa all habitats | Independent; Based at the University of Cambridge, UK | 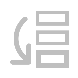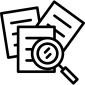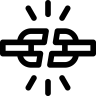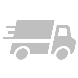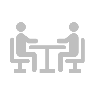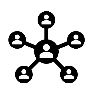 |
| [Electric Power Research Institute (EPRI)](https://www.epri.com/) | Farmers, landowners, water suppliers. Focus on electric power companies | Research; demonstration projects; webcasts, workshops, fact sheets, briefing notes | Primarily North America, but also some work internationally | 1970 | 900 employees, $450M/year | Grants, collaborations, members | Electricity generation, use, and involved stakeholders & natural resources | Independent nonprofit | 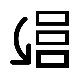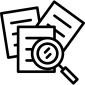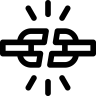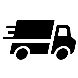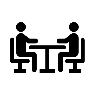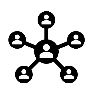 |
| [Foundations of Success, Inc.](https://fosonline.org/) | Conservation practitioners at any level, including NGOs, government agencies, donors & funders, conservation coaches, academics | One-on-one meetings or in multi-team workshop with conservation teams and organizations to determine what evidence is needed to support situation analysis, theories of change for key conservation actions as part of the adaptive management cycle; assemble and make use of this evidence; develop generic models that encapsulate collective knowledge about these topics e.g., software tool (Miradi); created several publicly accessible libraries of specific and generic theory-of-change pathways – e.g., Conservation Actions and Measures Library (CAML) | Global/  International | 2000 | Small: 20 staff members with a wide range of projects | Primarily fee-for-service, but approximately 10% of costs are covered via grants; excess revenue from fee-for-service re-invested to support mission-based unpaid work, e.g., CAML | Adaptive management and evidence-based practice | Independent 501(c)(3) nonprofit organization; founding member / secretariat for the Conservation Measures Partnership (CMP), a consortium of conservation organizations whose mission is to credibly assess and improve the effectiveness of conservation actions; independent of the conservation organizations they work with but have close ties to them | 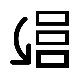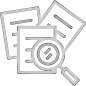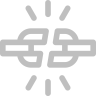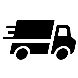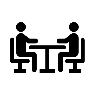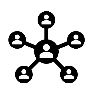 |
| [fRI Research](http://friresearch.ca/) | Government, industry (forestry, oil & gas, some coal), academia, NGOs | Peer-reviewed publications; land management tools; reports; research summaries; infographics; webinars; workshops | Alberta, British Columbia, Saskatchewan, and Northwest Territories, Canada | 1992 | 25-35 staff (seasonal variations), $5-6 million annually | Funded through shareholders, partners, and grants | Programs defined by partner needs; currently includes grizzly bears, caribou, mountain pine beetle, forest hydrology, healthy landscapes, migratory birds | Independent not-for-profit corporation and non-advocacy organization; Funded by industry, but focus is on science excellence | 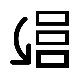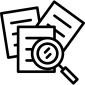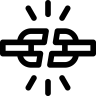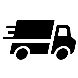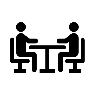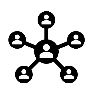 |
| [Great Lakes Acoustic Telemetry Observation System](https://glatos.glos.us/)  [(GLATOS)](https://glatos.glos.us/) | Primarily government resource managers and NGOs | Primarily journal articles and workshops; science transfer project working to develop a dashboard of GLATOS walleye movement data that managers can access | Great Lakes Basin (U.S. and Canada) | 2010 | >80 ongoing projects in the GLATOS network | Operate with grants from the Great Lakes Restoration Initiative as well as some funding from the Great Lakes Fishery Commission | Movement ecology of Great Lakes Fishes, particularly those of commercial fishing interest as well as invasive species | Primarily work with government and NGO partners; not much affiliation with industry | 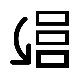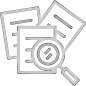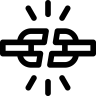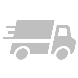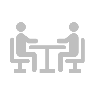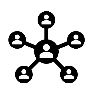 |
| [Great Lakes Fishery Commission](http://www.glfc.org/index.php) | Fishery Managers (primarily state, provincial, tribal/First Nations) | Journals; workshops; fact sheets, presentations; e-newsletters | Great Lakes Basin (U.S. and Canada) | 1955 | Staff approximately 25 people | Government appropriations, grants | Geographically focused; three pillars: sea lamprey control, cooperative fisheries management, science | Housed in Ann Arbor, MI; a treaty organization with certain communities; not affiliated with industry | 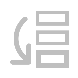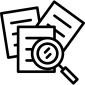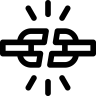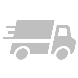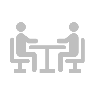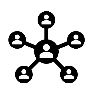 |
| [Miistakis Institute](http://rockies.ca/) | Agricultural producers, landowners, governments (municipal, provincial, federal), NGO's, industry, public | Peer-reviewed articles, reports, videos, webinars, briefing notes, fact sheets, databases, workshops | Alberta, Canada | 1997 | 6 full time staff, additional contractors for specific project work | Combination of grants (foundations, government grants) and fee-for-service | Transportation ecology, private land conservation, municipalities and conservation, citizen science for conservation, human wildlife coexistence and conservation planning and policy | Completely independent organization; hold an affiliation with Mount Royal University and are housed there | 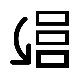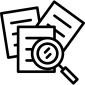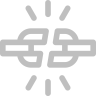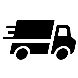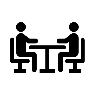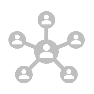 |
| [National Center for Ecological Analysis & Synthesis (NCEAS)](https://www.nceas.ucsb.edu/) | Nonprofit organizations; government agencies, and foundations | 'Working groups': teams of researchers and, sometimes, practitioners from agencies and organizations who translate the science into on-the-ground solutions; fund mathematical and geospatial models; peer-reviewed publications | Global/  International | 1995 | 40 active projects and employs 93 people; approximately $44 million in extramural funding | Costs covered by federal, state and private funding | Thematically focused: core ecology, ecoinformatics, conservation and resource management | Organized Research Unit through the University of California, Santa Barbara. | 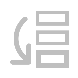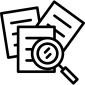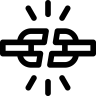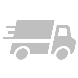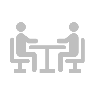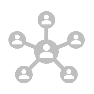 |
| [National Environmental Science Program Threatened Species Recovery Hub](http://nespthreatenedspecies.edu.au/) | Government natural resource managers; Species, conservation and parks managers; NGOs; Traditional owners; non-government natural resource management agencies; primary industries government agencies (water, ag, forests, fisheries); landcare (community) conservation groups; city councils; farmers; industry proponents; urban developers | Co-delivery of projects with partners; meetings; workshops; reports; fact sheets; emails; articles in relevant magazines; media; social media; videos; showcases; extension and farmer field days; presentations and industry events and conferences | Australia | 2015 | 160 projects, ~200 research, students and support staff, 250+ partner organisations, $60m over 5 years | Government grant with matched funding ($ and in-kind) from research agencies, a lot of co-investment and in-kind from partners | Terrestrial, freshwater and coastal biodiversity; priorities are broadly set by lead funding agency (Australian Government Department of Agriculture, Water and the Environment), with annual research planning co-designed between hub and lead funding agency, and informed by key stakeholders through a Stakeholder Reference Group | Housed in research agencies (primarily universities); government is the primary co-design partner and industry partners are collaborating partners on many projects, but the program is governed by principles of academic freedom and research independence | 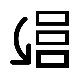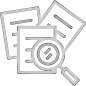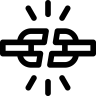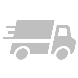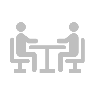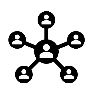 |
| [National Council for Air and Stream Improvement (NCASI)](https://www.ncasi.org/) | Forest products industry | Technical bulletins; special reports; peer-reviewed journal articles; briefing notes; fact sheets; white papers; webinars; workshops; infographics | Canada and U.S. | 1943 | Approximately 60 staff | Membership Dues | Environmental and sustainability topics, including analytical chemistry, chemical engineering, paper science, computer modeling and simulation, statistics, toxicology, forest biology, forest ecology, wildlife biology and aquatic biology | Funded by industry, but focus is on science excellence | 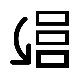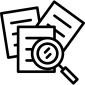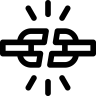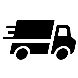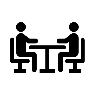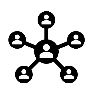 |
| [NatureServe Canada](http://www.natureserve.ca/) | All practitioners | Online data portal (NatureServe Explorer), custom data requests, reports | Canada (but international NatureServe Network is focused on western hemisphere) | 1999 | Annual revenues approximately 2 million. 5 full-time staff. Most funds allocated to member organizations for projects | Primarily grants and contributions from Canadian government; increasing revenue stream from foundations and other NGOs | Rare and threatened species (what, where, how are they doing, threats). Small but growing focus/capacity on ecosystems. | Organization is independent but 95% of its member organizations are provinces, territories, government | 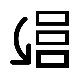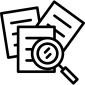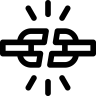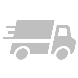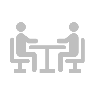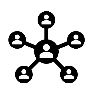 |
| [Nebraska Extension](https://extension.unl.edu/) | Landowners, farmers, ranchers | Workshops; publications; news articles | Nebraska, U.S. | 1918 | 300 Faculty educators | Fees, state or federal dollars, grants | Agriculture and natural resources | Housed at the University of Nebraska–Lincoln | 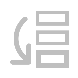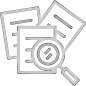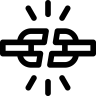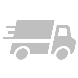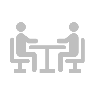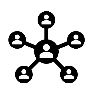 |
| [Northern Australia Environmental Resources Hub](http://nespnorthern.edu.au/) | Primarily state and federal government decision-makers, and on-the-ground land managers | In-person briefings; workshops; factsheets; website and social media; reports; data sets; videos; and scientific papers | Northern Australia | 2016 | The Hub is funded by AUD24 million+ from the Australian Government;  40+ research projects; 100+ researchers | Funded by the Australian Government’s National Environmental Science Program with in-kind contributions from research partner institutions | Geographically focused on the wet-dry tropics of northern Australia; the hub theme is to provide world-class research to support the sustainable development of northern Australia | Housed at Charles Darwin University in the Northern Territory, Australia. Funding comes from the federal government with in-kind contributions from research institutions, not industry | 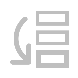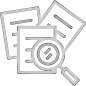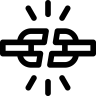 |
| [Pepperwood](https://www.pepperwoodpreserve.org/) | Government resource managers, NGOs, other | Journals; workshops; fact sheets | Northern California, U.S. | 2010 | 19 members of staff | Donations, grants, and some fee-for-service | California geography; climate, vegetation, and wildlife topics | Independent nonprofit |  |
| [Rights-of-Way as Habitat Working Group](http://rightofway.erc.uic.edu/) | Energy companies and transportation agencies, government organizations, conservation groups and non-profits, private landowners, academic institutions, and contractors | Bi-annual workshops; webinars and video tutorials; online discussion board; online resource library with case studies and best management practices; tools (e.g., pollinator habitat scorecard), and email communications | Canada and U.S. | 2015 | Primarily 1.5 staff, 2 interns | Primarily grant funded; some industry funding | Pollinator habitat on energy and transportation rights-of-way, taking into account endangered species regulations, habitat evaluation and monitoring, and educating and collaborating on resource sharing and best management practices | Energy Resources Center, University of Illinois Chicago |  |
| [Society for Ecological Restoration](https://www.ser.org/) | Ecological restoration practitioners and restoration ecologists | Newsletters; publications. scientific journal *Restoration Ecology*; the Restoration Resource Center; webinars | Global/  International | 1988 | Over 3000 members, over ten staff members | Charitable sponsorship and member dues | Ecological restoration | Housed in Washington, DC. but run remotely by staff; accept donations and sponsorships from some industry partners, but are not affiliated with industry |  |
| [Southern Cape Landowners Initiative](https://www.scli.org.za/) | Landowners, scientists | Workshops, blog | South Africa | 2011 | Small | Grants | Ecosystem management | Independent |  |
| [Stockholm University Baltic Sea Centre (formerly the Baltic Eye project)](https://www.su.se/ostersjocentrum/english/) | Decision-makers (Swedish politicians, EU politicians); practitioners (e.g., fishermen, farmers, NGOs, government resource managers, municipal officials) | Seminars arranged on specific topics, targeted for the audience; policy briefs and fact sheets; web articles spread in social media and newsletters; workshops; personal contacts and meetings; exhibitions and close cooperation with Skansen Baltic Sea Science Center, Sweden's largest museum | Primarily Sweden, but the entire Baltic region | 2013 | Approximately 40 people working at the Stockholm University Baltic Sea Centre; 52 million SEK 2018 | Foundation and Stockholm University funding, grants | Habitats and biodiversity, sustainable fisheries, eutrophication, and pollutants | A centrum at Stockholm University and are thus totally independent from the industry |  |
| [UC Agriculture and Natural Resources](https://ucanr.edu/) | Landowners, farmers, ranchers, foresters, government and commodity industries | Cal Ag journal, blogs, webinars, videos, workshops, fact sheets | California, U.S. | 1914 | 1500 employees throughout California | County, State and Federal funds, grants, donations and program revenue | Agriculture & pest management; environment & natural resources; food & health etc. | Housed at the University of California |  |
| [The Western Australian Biodiversity Science Institute](https://wabsi.org.au/) | Industry, government regulators and resource managers | Workshops; reports; tools, templates, frameworks | Western Australia | 2015 | Small executive team of 7 working on strategic projects for the state of Western Australia | State funded with some private co-investment | Research priorities based on the needs of end users from industry and government, through extensive stakeholder engagement | Independent joint venture of leading research organisations in the State |  |
| [Yellowstone to Yukon Conservation Initiative](https://y2y.net/) | Conservation groups, local landowners, businesses, government agencies, Native Americans and First Nations, scientists | Listserv and weekly newsletter sharing recent scientific papers/reports along with a short synopsis of what it means and why it matters; briefing notes to government staff and elected representatives; workshops; webinars with invited experts; guest lectures; public talks; writing reports; participating in roundtables, expert panels | Yellowstone (U.S.) to Yukon (Canada) region | 1993 | Currently ~30 staff (full- and part-time) and $4M USD in revenue for 2019 | Grants, donations | Geographically focused on the Yellowstone to Yukon region; thematically focused on ecological connectivity and wildlife conservation | Headquarters in Canmore, Alberta, Canada with staff across the Y2Y region (in US and Canada); no affiliation with industry, little funding from industry |  |
